# Guidelines or guidance for better idiopathic pulmonary fibrosis management?

**DOI:** 10.1186/s12916-016-0567-9

**Published:** 2016-02-10

**Authors:** Jürgen Behr

**Affiliations:** Department of Internal Medicine V University of Munich and Asklepios Clinic München-Gauting, Comprehensive Pneumology Center Munich, Member of the German Centre for Lung Research, München, Germany

**Keywords:** Evidence-based medicine, GRADE, Guidelines, Idiopathic pulmonary fibrosis

## Abstract

Idiopathic pulmonary fibrosis (IPF) is a rare fibrotic interstitial lung disease with a relentlessly progressive course and fatal outcome. Guidelines summarizing the current evidence and providing evidence-based recommendations for the treatment of rare diseases such as IPF are important since individual physicians often have limited experience. Nevertheless, the available evidence is often scarce and, therefore, evidence-based recommendations are prone to being vague or with low confidence, thus creating uncertainty instead of guidance. Moreover, the effect of guidelines themselves on clinical practice has not been sufficiently evaluated. On the other hand, expert opinion may be biased and lead to the misinterpretation of evidence, resulting in misleading recommendations and a potential harm to patients. This editorial focuses on the advantages and disadvantages of evidence-based guidelines and professional experience in the context of a rare disease such as IPF and tries to assess the optimum combination of both approaches.

Please see related commentary articles: http://dx.doi.org/10.1186/s12916-016-0562-1 and http://dx.doi.org/10.1186/s12916-016-0563-0

## Guidelines and evidence-based medicine: the downfall of the Holy Grail

Over 20 years ago, evidence-based medicine began to replace medical practices based on tradition, anecdotes, and theoretical reasoning through empirical evidence from clinical trials [1]. The foundations of evidence-based medicine rely on the development and implementation of evidence-based guidelines. Ideally, the knowledge obtained from high-quality clinical trials should translate directly into patient management in daily practice, thus improving patient care and replacing unproven clinical approaches. Unfortunately, this courageous concept has several inherent problems. Given the extremely high cost of good-quality clinical trials, these are mostly performed under the direction of the pharmaceutical industry, thus targeting their needs for the approval of new therapies rather than the often differing needs of clinical practice [1]. Consequently, many challenges faced in daily patient care have never been nor will ever be addressed through an evidence-based approach. Moreover, guidelines based solely on evidence from clinical trials are prone to overestimated, standardized diagnostic procedures in accordance with clinical trial needs, thus leading to a management-centered rather than a patient-centered approach [2]. Inflexible rules from evidence-based guidelines also exclude a significant proportion of the patient population from an appropriate clinical diagnosis and potentially disenfranchise them from available therapies, leaving patients and physicians in a diagnostic and therapeutic void and increasing diagnostic and therapeutic uncertainty [2]. Finally, complex diseases and multimorbidity are difficult to address with evidence-based guidelines, since clinical trials tend to exclude complexity for evident reasons. Linked to this is the challenge of the methodology of guideline development. Experience with the Grading of Recommendations, Assessment, Development and Evaluations (GRADE) methodology reveals that the recommendations are sometimes insufficient to allow an appropriate differentiation. Moreover, the wording is often awkward, using formulas which make it difficult for physicians to fully understand the meaning of a recommendation [3, 4]. Conversely, expert opinion based on clinical experience may be able to provide clear guidance even in the absence of formal evidence. Regrettably, in the past, opinion hegemony of powerful personalities or groups, as well as supposed “evidence-based medicine”, were the source of mis- or over-interpretation of the available evidence eventually leading to biased, potentially harmful recommendations. The above issues are of particular importance in a rare disease such as idiopathic pulmonary fibrosis (IPF), where individual physicians often have limited experience and rely on the available guidance.

IPF treatment guideline development is an archetypal example to examine the positive and negative impacts of evidence-based guidelines. The 2011 ATS/ERS/JRS/ALAT guideline is an excellent summary of the available evidence and provides and implements a new definition of IPF [3]. By upgrading the utility of high-resolution computed tomography (HRCT) and defining the radiological usual interstitial pneumonia pattern, the guideline also significantly changed the diagnostic process, eliminating the need for a surgical lung biopsy for patients with a definite usual interstitial pneumonia pattern on HRCT [5]. Nevertheless, interpretation of HRCT is often not unequivocal, and thus diagnostic uncertainty has increased in many patients [2, 5]. A surgical lung biopsy is performed to rule out uncertainty; however, it is not feasible for a significant number of patients with IPF due to disease severity, comorbidities, frailty, or cost [5]. The multidisciplinary discussion proposed as the gold standard in the guideline leads to a choice of various diagnostic probabilities – definite, probable, and possible; however, the therapeutic implications are defined only for the definite diagnostic category of IPF. The most recent update of the IPF guideline focused on disease treatment and recommended the two available drugs, nintedanib and pirfenidone, for the majority of IPF patients [6]. However, evidence-based IPF guidelines do not address the treatment of patients with a “probable” or “possible” IPF diagnosis [6]. In this context, it is also imperative to comprehend that guidelines themselves are an intervention in clinical practice, with both potentially beneficial and harmful consequences. Despite this, the net effect of guidelines after their implementation remains to be addressed.

## The art of clinical decision making

As indicated by Rochwerg et al. [7], evidence-based guidelines provide a comprehensive summary of the available evidence and a transparent process leading to recommendations which are not biased by personal experiences or opinions of individual experts. The strength of the GRADE methodology used in this process lies on the systematic and pragmatic literature search which leads to an unbiased overview of the available evidence in a given topic. Its shortcoming is the fact that the methodology tends to exclude any clinical experience and common sense not supported by formal evidence from well-designed clinical trials, as succinctly discussed by Wells [8]. This approach eventually leads to recommendations which lack practicability, as exemplified in the diagnostic algorithm of the IPF guideline, which foresees a surgical lung biopsy for patients with a non-informational HRCT, but yet this cannot be performed in a large portion of these patients due to disease severity, comorbidities, or cost. Consequently, according to this guideline, a significant number of patients is left without a diagnosis or treatment [3, 7]. This differentiation has gained significance since the recent approval of nintedanib and pirfenidone [6], since the issue of whether these drugs should also be used in probable and possible IPF remains unresolved. While possible IPF patients have been included in the INPULSIS trials with nintedanib, only definite IPF patients were allowed in the ASCEND trail with pirfenidone [7–10]. However, neither of these studies has led to a definite solution. In the absence of clinical trials addressing this question, an evidence-based recommendation will not be possible; nevertheless, for clinical guidance, a practical recommendation is highly desirable. Therefore, complementing evidence-based recommendations with clinical expertise and advice in areas with little or no evidence should be considered in order to provide practical guidance. There is no doubt that the 2011 evidence-based IPF guidelines [3] are appropriate for the diagnosis of approximately 70 % of IPF cases; however, there is no guidance regarding the diagnosis and treatment of the remaining 30 % for whom the suggested alternative diagnostic test is not feasible. Thus, rigid interpretation of guidelines may be deleterious for patients by excluding them from appropriate diagnosis and therapy. The evidence-based guideline approach overemphasizes standardized diagnostic tools and neglects the art of clinical decision making. The latter takes into consideration all available information on an individual patient, including the clinical behavior of the disease and response to previous therapies, and is of paramount importance for those patients who fall beyond the evidence base. A multidisciplinary discussion, including pneumologists, radiologists, and pathologists, to decide on an individual patient basis, as proposed in the guideline, appears to be the optimal setting in which to practice this art of clinical decision making.

## Conclusions

Evidence-based guidelines summarize the available evidence using a comprehensive literature research approach and provide unbiased recommendations reflecting this evidence. However, this emphasis on methodology eventually results in a lack of practicability and guidance in areas with a dearth of evidence. The empirical approach based on expert advice can provide guidance, especially in areas with little or no formal evidence, but is vulnerable to biased recommendations. Thus, both approaches may adversely affect patients. A synthesis of these potentially complementary approaches seems appropriate to provide optimal guidance in the treatment of all patients.

## Abbreviations

GRADE: Grading of Recommendations, Assessment, Development and Evaluations
HRCT: High-resolution computed tomography
IPF: Idiopathic pulmonary fibrosis
